# Supplementary material for: Interferon‐beta signaling in retinal mononuclear phagocytes attenuates pathological neovascularization
Source: EMBO Mol Med. 2016 May 3;8(6):670–8. doi: 10.15252/emmm.201505994 (PMC4888856; doi:10.15252/emmm.201505994)
Supplement: Supplementary file 1 — Appendix [file EMMM-8-670-s001.pdf]

Appendix

Interferon-beta signaling in retinal mononuclear phagocytes attenuates pathological neovascularization

Anika Lückoff, Albert Caramoy, Rebecca Scholz, Marco Prinz, Ulrich Kalinke and Thomas Langmann

Table of contents **Page**

Appendix Figure S1	2
Appendix Figure S2	3
Appendix Figure S3	4
Appendix Supplementary Materials and Methods	6
Appendix References	8

Appendix Figure S1.

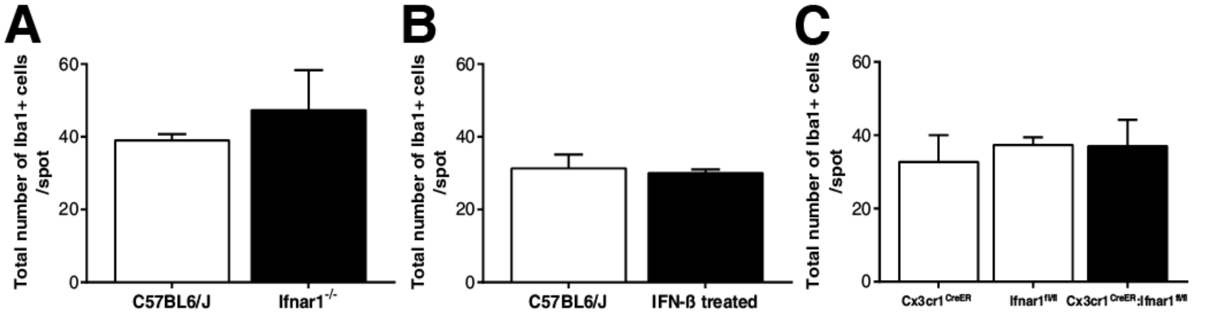

Appendix Figure S1. Total number of Iba1+ cells within laser spots. The total numbers of Iba1+ cells were counted within a circular region of 200 µm diameter around the laser spot, indicating no significant differences between the respective groups (Ifnar1$^{-/-}$ (**A**), IFN-ß treatment (**B**) and Cx3cr1^{CreER}:Ifnar1$^{flox/flox}$ (**C**)). Values show mean ± SD. (n = 3 spots from different retinas).

Appendix Figure S2.

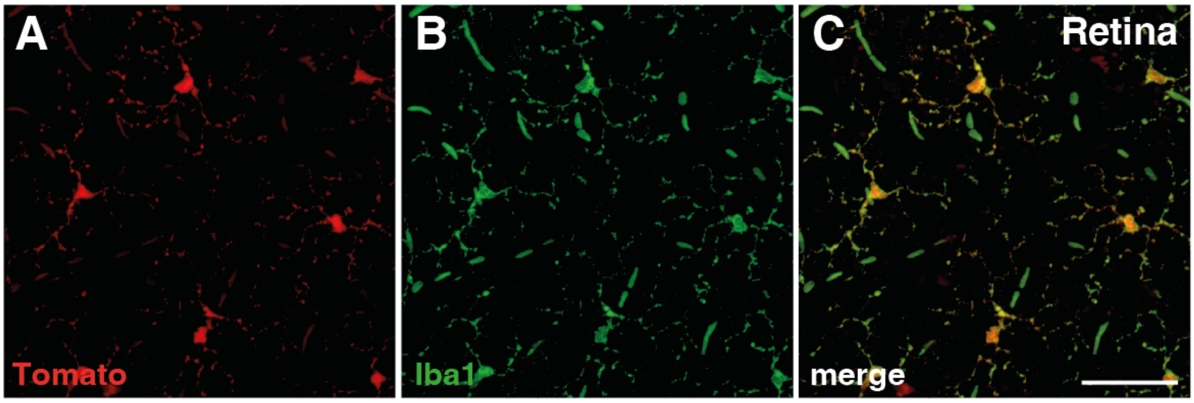

Appendix Figure S2. Cre activity in retinal microglia. Representative images of retinal flat mounts of Cx3cr1^{CreER}:R26^{tomato} mice co-stained with Iba1 four weeks after tamoxifen injection showing microglia specificity of the Cx3cr1-promotor controlled recombinase. The co-localization of the tomato signal (**A**) with the Iba1 staining (**B**) indicates high recombination efficiency (**C**). Scale bar 50 µm.

Appendix Figure S3.

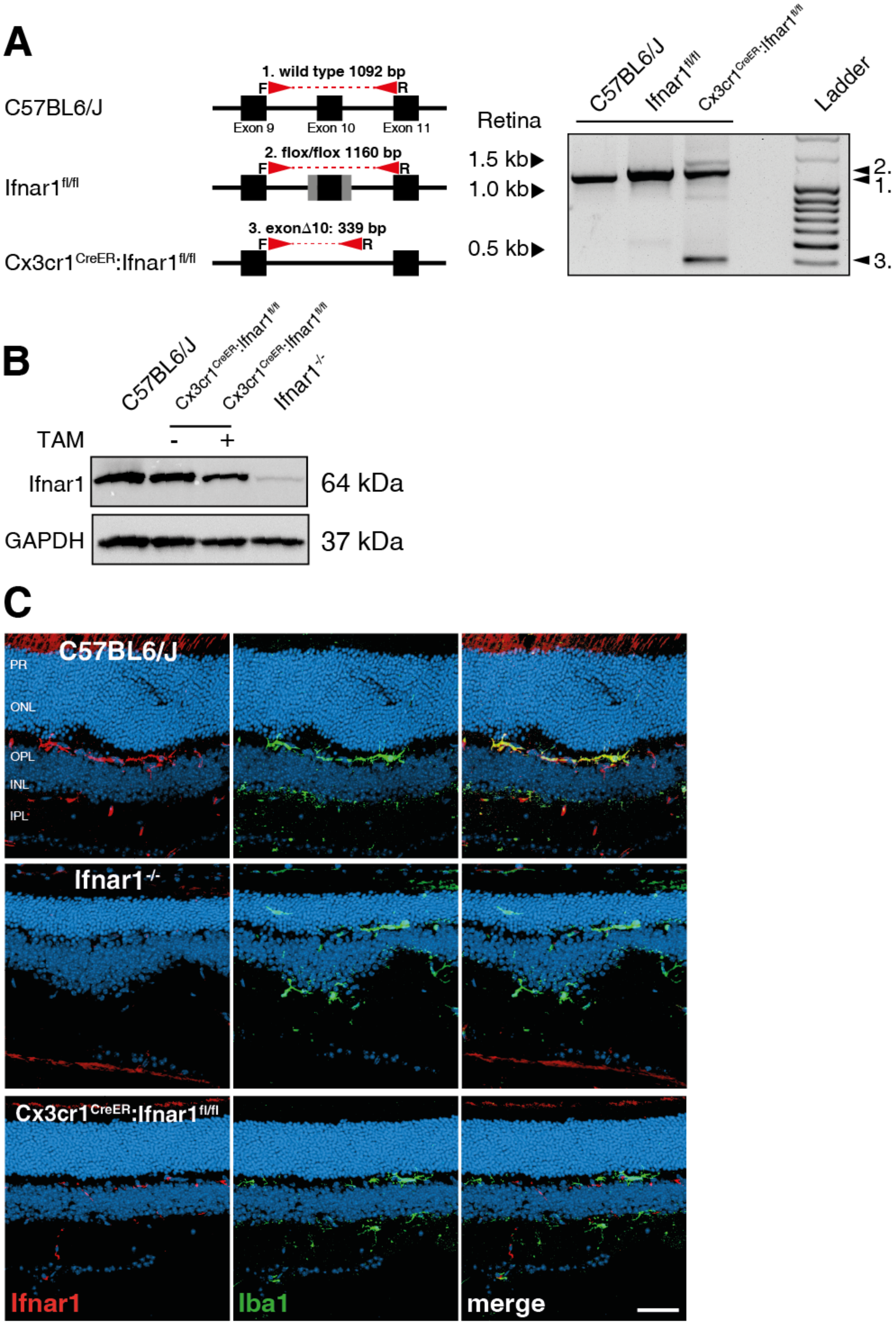

Appendix Figure S3. Knock down of Ifnar1 in Cx3cr1^{CreER}:Ifnar1$^{flox/flox}$ mice. Exon structure of the Ifnar1 gene locus is schematically shown of C57BL6/J wild type, Ifnar1$^{flox/flox}$ and tamoxifen treated Cx3cr1^{CreER}:Ifnar1$^{flox/flox}$ mice. To confirm the conditional knockout of *Ifnar1*, PCR analyses were performed with genomic DNA from retina samples. Amplification of a floxed exon 10 resulted in a 1160 bp fragment, while the excision of exon 10 (Δ10) leads to a 339 bp fragment (**A**). A Western blot confirmed a reduced *Ifnar1* expression in retinal protein extracts of tamoxifen-treated Cx3cr1^{CreER}:Ifnar1$^{flox/flox}$ mice (**B**). Representative images of retinal cross sections of C57BL6/J, Ifnar1$^{-/-}$ and Cx3cr1^{CreER}:Ifnar1$^{flox/flox}$ mice co-stained with antibodies against Iba1 and Ifnar1 revealed a weakened Ifnar1 signal in Cx3cr1^{CreER}:Ifnar1$^{flox/flox}$ cryo sections (**C**).

Appendix Supplementary Materials and Methods

Immunohistochemistry of retinal cross sections

For immunohistochemistry 14 µm cryo slices from eyes embedded in OCT (Tissue-Tec®, Sakura) were blocked (1% dry milk powder, 0.01% Tween20 in 1x PBS) for 30 min and stained with primary antibody Iba1 (1:500; 019-19741, Wako) or IFN-αR1 (1:500; MAR1-1H5, sc-53590 Santa Cruz) over night at 4°C followed by incubation with the secondary antibody goat anti-rabbit (1:1000; A11012, Thermo Scientific) or goat anti-mouse (1:1000, A11005, Thermo Scientific) for 1 hour. Images were acquired with an Imager.M2 microscope equipped with an ApoTome.2 (Carl Zeiss).

Dextran labeling of choroidal neovascularization

To stain the choroidal neovascularization 200 µl FITC labeled dextran (FD-250S, Sigma-Aldrich) was injected intravenously 5 min before sacrificing the mice (Lambert, Lecomte et al., 2013). After additionally perfusing the animals with 5 mg/ml FITC-dextran in 10 ml PBS (Semkova, Peters et al., 2003) the eyes were enucleated and fixed in 4% PFA. For lectin co-staining retinal pigment epithelia/choroidal samples were incubated in TRITC-conjugated lectin for 1h (L5264, Sigma-Aldrich) (Luhmann, Lange et al., 2012).

Cell culture

The murine microglia-like cell line BV2 (Blasi, Barluzzi et al., 1990) was cultured in RPMI 1640 medium (31870, Gibco®) mixed with 2 mN L-glutamin (25030-024, Gibco®), 1% Penicillin/Streptomycin (15140-122, Gibco®), 5% FCS (10270-106, Gibco®) and 0.01% ß-Mercaptoethanol and the human microglia-like cell line SV40 (Reiner, Heldt et al., 2015) in DMEM High Glucose medium (D5796, Sigma-Aldrich) mixed with 10% FCS and 10% Penicillin/Streptomycin at 37°C and 5% CO_2 humidity. The cells were regularly tested for mycoplasma contamination with the MycoAlert® kit (LT07-418, Lonza). For IFN-ß stimulation cells were transferred into a 6-well plate and stimulated with 1000 U/ml human (300-02BC, PeproTech) or murine IFN-ß (IF011, Millipore) for 24 hours.

RNA isolation and quantitative real time PCR (qRT-PCR)

RNA was isolated using the NucleoSpin® RNA Mini Kit (740955, Macherey-Nagel) and RNA quantity was measured spectrophotometrically with a NanoDrop 2000 (Thermo

Scientific). Reverse transcription was carried out with the RevertAid RT Kit (K1691, Thermo Scientific).

For qRT-PCR the TaqMan 7900HT PCR detection system (Invitrogen Life Technologies) was used with 10 µl reaction mixture containing 50 ng cDNA, 1x TaqMan Gene Expression Master Mix (Invitrogen Life Technologies), 200 nM primers and 0.25 µl dual-labeled probe (Roche Universal Probe Library, Roche). Following genes, primers and probes have been used: human MX1 forward, 5'-ttcagcacctgatggccta-3' and revers, 5'-aaagggatgtggctggagat-3' primer (probe #79), murine Mx1 forward 5'-ttcaaggatcactcatacttcagc-3' and revers, 5'-gggaggtgagctcctcagt-3' primer (probe #53), murine Mx2 forward, 5'-tgcggttgtgagcctctt-3' and revers, 5'-tgcggttgtgagcctctt-3' primer (probe #11), human GAPDH forward, 5'-gcccaatacgaccaaatcc-3' and revers, 5'-agccacatcgctcagaca-3' primer (probe #60) and murine Atp5b forward, 5'-ggcacaatgcaggaaagg-3' and revers, 5'-tcagcaggcacatagatagcc -3' primer (probe #77). The PCR reaction parameters were 10 min 95°C, 40 cycles of 15 s 95°C melting and 1 min 60°C annealing/extension. The results were analyzed using the ΔΔCt method for relative quantification.

DNA from Retina samples/ PCR of Δ10

The PCR analyses were performed with primers located upstream (5'-ggttaagctccttgctgctatctgg-3') and downstream (5'-ttggagatgcaatctgctactcagc-3') of the loxP-flanked exon 10 of the Ifnar1 gene (Detje, Meyer et al., 2009). Using genomic DNA isolated from retina samples (DNeasy Blood & Tissue Kit, 69504, Qiagen), PCR amplification of DNA from C57BL6/J and Ifnar1$^{flox/flox}$ mice resulted respectively in a 1092 bp and 1160 bp fragment containing exon 10. In contrast, the retinal DNA of Tamoxifen-treated Cx3cr1^{CreER}:Ifnar1$^{flox/flox}$ mice displayed an amplicon of 1160 bp and 339 bp.

Western Blot

Retinal tissue was homogenized in ice-cold RIPA buffer (150 mM Sodium chloride, 1% NP-40, 0.5% Sodium deoxycholate, 0.1% SDS, 50 mM Tris-HCl pH 7.4 in deionized water) using a TissueLyser LT (Qiagen) and centrifuged at 4°C for 5 min at 5000 g. The protein concentrations were determined by Bradford assay (Roti®-Quant, K015.1, Roth) according to the manufacture's protocol. 30 µg of protein were separated by SDS-PAGE on a 10% gel using the PageRuler Prestained Protein Ladder (#26616, Thermo Scientific). Proteins were transferred to a 0.45 µm nitrocellulose membrane (#9004-70-0, Bio-RAD) and blocked in 5% milk powder in 1x PBS for 1 hour. The membrane was incubated with the primary antibody

IFN-αR1 (1:500; MAR1-1H5, sc-53590 Santa Cruz) or GAPDH (1:1000; I-19; sc-48166, Santa Cruz Biotechnology) overnight at 4°C and with the secondary antibody goat anti-mouse IgG-HRP or rabbit anti-goat IgG-HRP (1:2500; sc-2005/sc-2768, Santa Cruz Biotechnology) for 1 hour at room temperature. The western blot signal was visualized using SuperSignal West Pico (#34077, Thermo Scientific) to oxidate the horseradish peroxidase and the Multiimage II system (Alpha Innotechnology) to detect chemiluminescence.

Appendix References

Blasi E, Barluzzi R, Bocchini V, Mazzolla R, Bistoni F (1990) Immortalization of murine microglial cells by a v-raf/v-myc carrying retrovirus. Journal of neuroimmunology 27: 229-37

Chen M, Zhao J, Luo C, Pandi SP, Penalva RG, Fitzgerald DC, Xu H (2012) Para-inflammation-mediated retinal recruitment of bone marrow-derived myeloid cells following whole-body irradiation is CCL2 dependent. Glia 60: 833-42

Detje CN, Meyer T, Schmidt H, Kreuz D, Rose JK, Bechmann I, Prinz M, Kalinke U (2009) Local type I IFN receptor signaling protects against virus spread within the central nervous system. J Immunol 182: 2297-304

Lambert V, Lecomte J, Hansen S, Blacher S, Gonzalez ML, Struman I, Sounni NE, Rozet E, de Tullio P, Foidart JM, Rakic JM, Noel A (2013) Laser-induced choroidal neovascularization model to study age-related macular degeneration in mice. Nat Protoc 8: 2197-211

Luhmann UF, Lange CA, Robbie S, Munro PM, Cowing JA, Armer HE, Luong V, Carvalho LS, MacLaren RE, Fitzke FW, Bainbridge JW, Ali RR (2012) Differential modulation of retinal degeneration by Ccl2 and Cx3cr1 chemokine signalling. PLoS One 7: e35551

Reiner A, Heldt SA, Presley CS, Guley NH, Elberger AJ, Deng Y, D'Surney L, Rogers JT, Ferrell J, Bu W, Del Mar N, Honig MG, Gurley SN, Moore BM, 2nd (2015) Motor, visual and emotional deficits in mice after closed-head mild traumatic brain injury are alleviated by the novel CB2 inverse agonist SMM-189. International journal of molecular sciences 16: 758-87

Semkova I, Peters S, Welsandt G, Janicki H, Jordan J, Schraermeyer U (2003) Investigation of laser-induced choroidal neovascularization in the rat. Invest Ophthalmol Vis Sci 44: 5349-54
