# Supplementary material for: Interferon‐beta signaling in retinal mononuclear phagocytes attenuates pathological neovascularization
Source: EMBO Mol Med. 2016 May 3;8(6):670–8. doi: 10.15252/emmm.201505994 (PMC4888856; doi:10.15252/emmm.201505994)
Supplement: Supplementary file 2 — Expanded View Figures PDF [file EMMM-8-670-s002.pdf]

Expanded View Figures

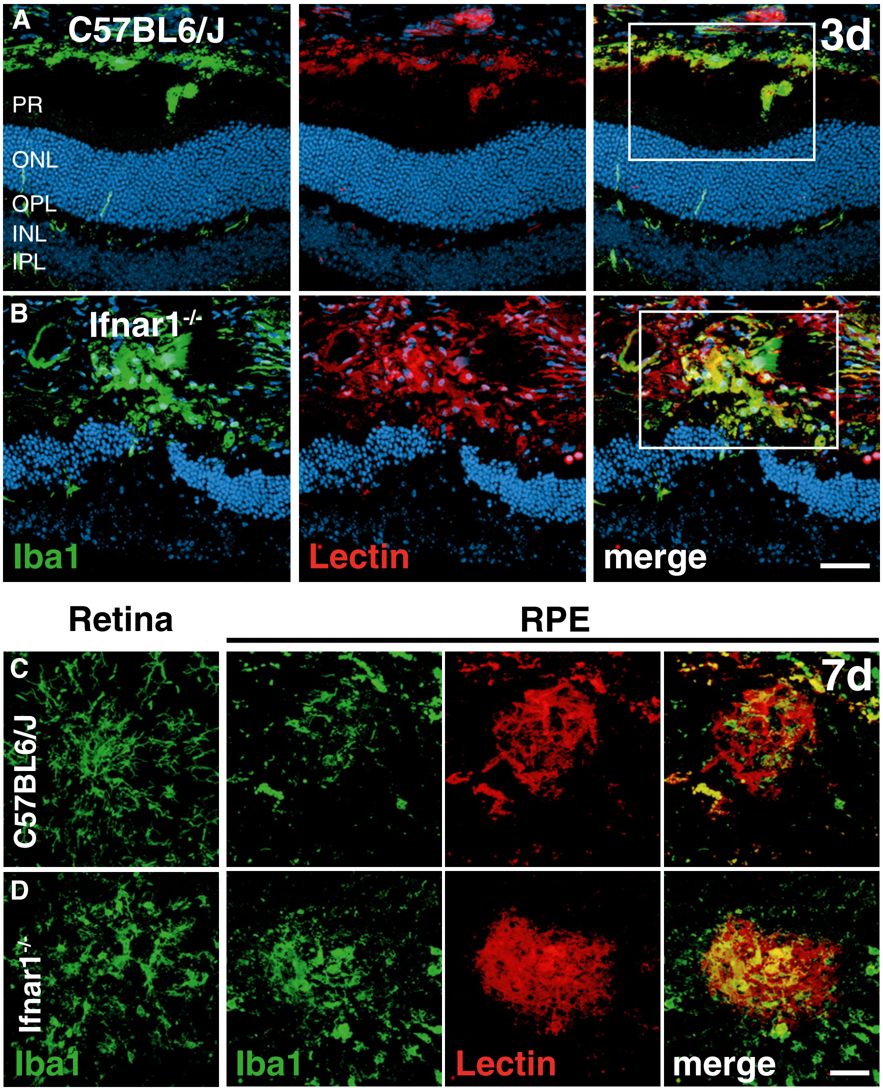

Figure EV1. Loss of Ifnar1 enhances CNV and leads to accumulation of mononuclear phagocytes in the subretinal space.

A, B Representative images of Iba1 (green) and lectin (red) co-stained retinal cross sections 3 days after laser coagulation in C57BL6/J controls (A) and *Ifnar1*$^{-/-}$ (B) mice with nuclear staining (DAPI, blue). The white box indicates the spot where the laser hit the retina. Scale bar 50 μm.

C, D Representative images of Iba1 (green) stained retinal flat mounts and Iba1 and lectin (red) co-stained RPE/choroidal flat mounts 7 days after laser coagulation in C57BL6/J (C) and *Ifnar1*$^{-/-}$ (D) mice. Scale bar 50 μm.

© 2016 The Authors

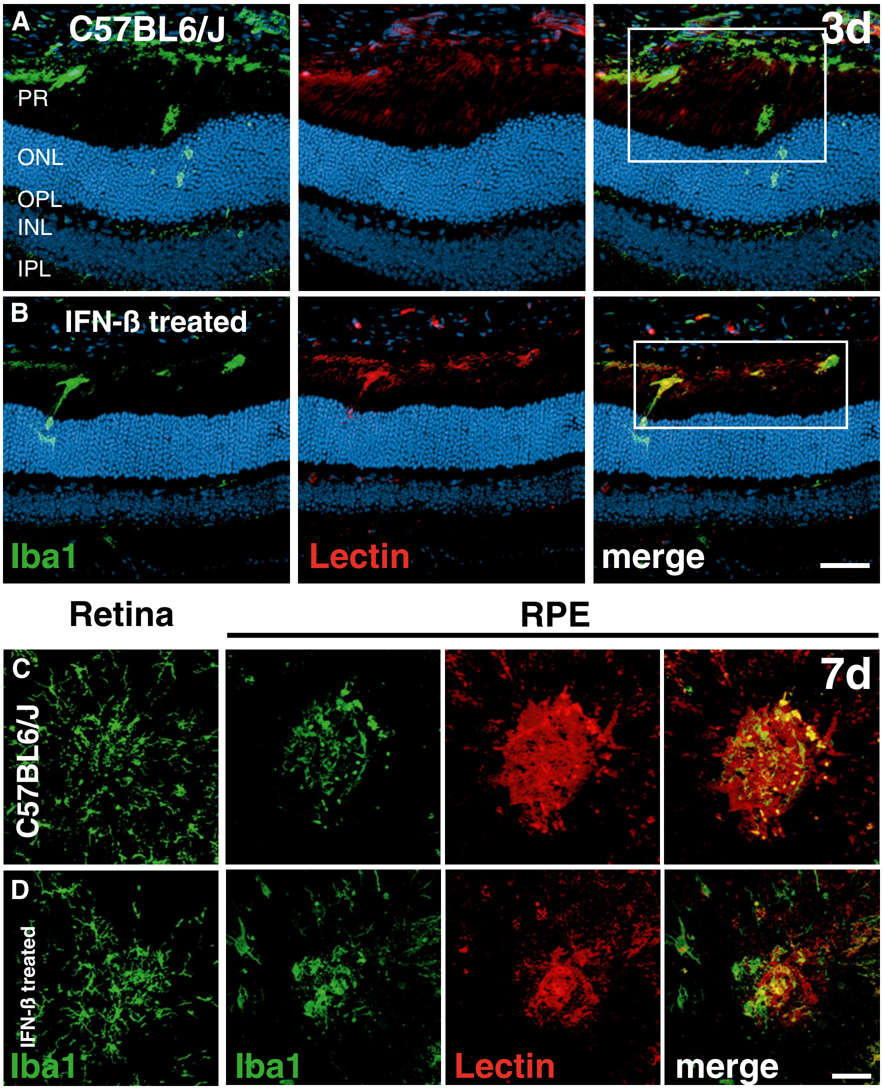

Figure EV2. Reduced CNV and subretinal mononuclear phagocyte accumulation in IFN-ß-treated mice.

A, B Representative images of Iba1 (green) and lectin (red) co-stained retinal cross sections 3 days after laser coagulation in C57BL6/J controls (A) and IFN-ß-treated C57BL6/J (B) mice with nuclear staining (DAPI, blue). The white box indicates the spot where the laser hit the retina. Scale bar 50 μm.

C, D Representative images of Iba1 (green) stained retinal flat mounts and Iba1 and lectin (red) co-stained RPE/choroidal flat mounts 7 days after laser coagulation in C57BL6/J controls (C) and IFN-ß-treated C57BL6/J (D) mice. Scale bar 50 μm.

 © 2016 The Authors

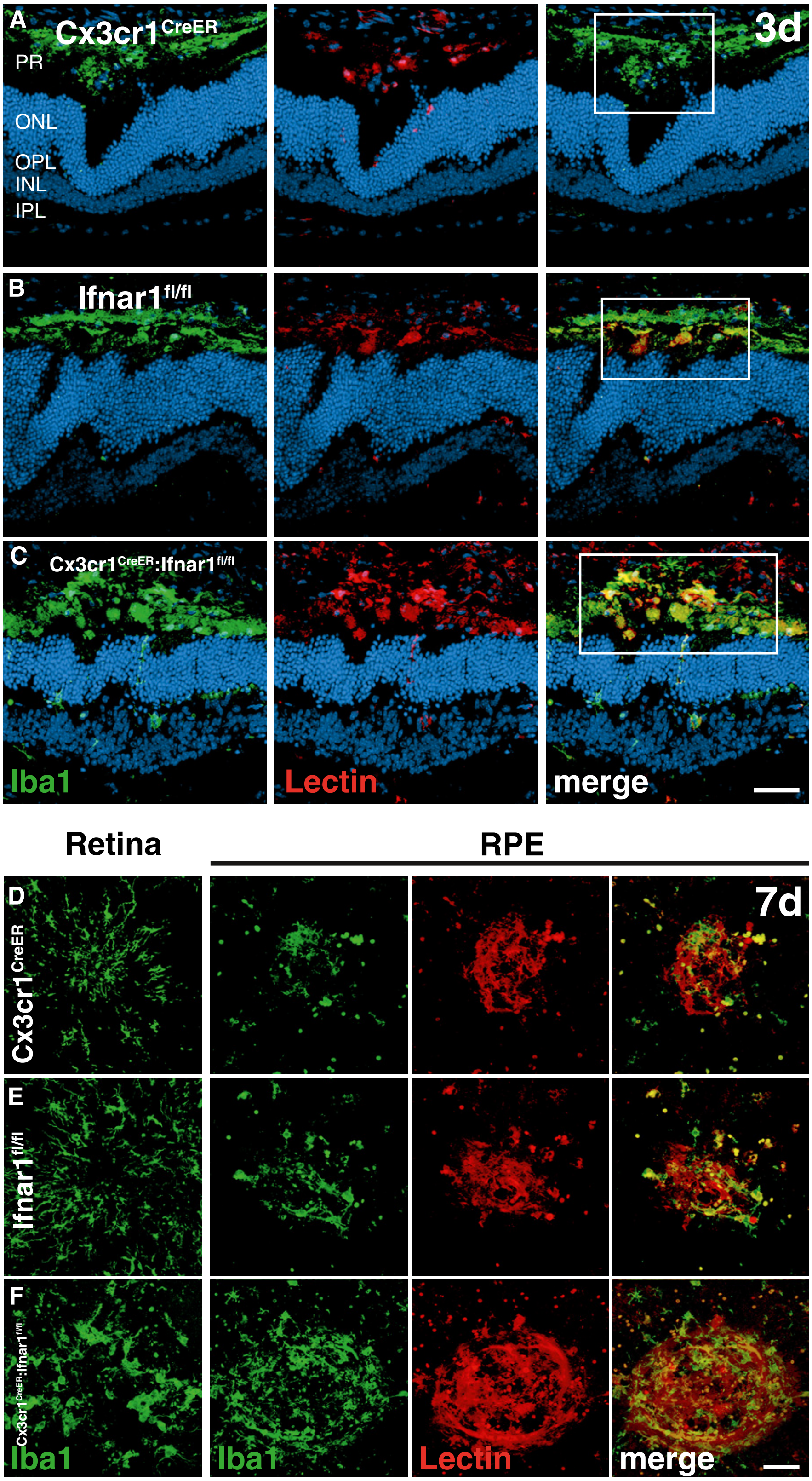

Figure EV3. Loss of Ifnar1 in microglia/macrophages leads to their subretinal accumulation and enhances CNV.

A–C Representative images of Iba1 (green) and lectin (red) co-stained retinal cross sections 3 days after laser coagulation in C57BL6/J controls (A) and *Ifnar1*$^{-/-}$ (B) and *Cx3cr1*^{CreER}:*Ifnar1*$^{fl/fl}$ (C) mice with nuclear staining (DAPI, blue). The white box indicates the spot where the laser hit the retina. Scale bar 50 μm.

D–F Representative images of Iba1 (green) stained retinal flat mounts and Iba1 and lectin (red) co-stained RPE/choroidal flat mounts 7 days after laser coagulation in C57BL6/J controls (D) and *Ifnar1*$^{-/-}$ (E) and *Cx3cr1*^{CreER}:*Ifnar1*$^{fl/fl}$ (F) mice. Scale bar 50 μm.

© 2016 The Authors
